# The *n*-Butanol Extract Obtained from the Inner Bark of *Tabebuia rosea* (Bertol.) DC, Specioside, and Catalposide Induce Leukemia Cell Apoptosis in the Presence of Apicidin

**DOI:** 10.3390/molecules29173986

**Published:** 2024-08-23

**Authors:** Nancy Yadira Guerrero-Pepinosa, Luz Angela Veloza, Juan Carlos Sepúlveda-Arias

**Affiliations:** 1 Grupo Infección e Inmunidad, Facultad de Ciencias de la Salud, Universidad Tecnológica de Pereira, Pereira 660003, Colombia; nancygue@unicauca.edu.co; 2 Facultad de Ciencias Naturales, Exactas y de la Educación, Programa de Biología, Universidad del Cauca, Popayán 190001, Colombia; 3 Grupo Polifenoles, Facultad de Tecnologías, Escuela de Química, Universidad Tecnológica de Pereira, Pereira 660003, Colombia; lveloza@utp.edu.co

**Keywords:** *Tabebuia rosea*, catalposide, specioside, apicidin, iridoids, cytotoxicity, apoptosis, cancer, antineoplastic agents

## Abstract

The cell signaling pathways involved in the antiproliferative activities of *T. rosea* inner bark remain unexplored. This study evaluated the apoptotic effects of two iridoids from the inner bark of *T. rosea* and apicidin on THP-1 cells. The cytotoxic effects of the extract and the pure compounds on THP-1 and Jurkat cells were also evaluated using the MTT assay. The apoptotic effect was determined by measuring the mitochondrial membrane potential. The expression of mRNA and MAPK kinase, Bax, and Bcl-2 proteins was detected by Western blotting and RT–qPCR, respectively. The extract and the compounds evaluated increased the percentage of apoptotic cells. Depolarization of the mitochondrial membrane was observed, and the number of cells in the G0/G1 phase increased. Catalposide and specioside significantly increased p38 protein expression, mostly in cells pretreated with apicidin. The p38 MAPK signaling pathway is at least one of the pathways by which the *n*-butanol extract obtained from *Tabebuia rosea*, catalposide, and specioside exerts its apoptotic effect on THP-1 cells, and this effect generates a response in the G0/G1 phase and subsequent cell death. In addition, there was depolarization of the mitochondrial membrane, an effect that was related to the participation of the proapoptotic protein Bax.

## 1. Introduction

Currently, cancer is a significant public health problem in terms of morbidity and mortality in developing countries; leukemia is a frequent cancer type and is among the fifteen most common news cases and deaths [1,2]. The most common cancer in children is leukemia, accounting for 30% of cases [3]. Worldwide, in children under 15 years of age, cancer does not represent more than 3% of all malignancies, but it has high mortality rates, especially acute pediatric leukemia [2]; this cancer is characterized by an increase in several abnormal white blood cells that can be either lymphoid or myeloid in origin. Chemoresistance in cancer cells and nonselective cytotoxic effects are troublesome treatments because they decrease the quality of life of leukemia patients. Therefore, research into novel therapeutic agents is essential for effectively treating leukemia.

Ethnopharmacological knowledge about the use of plants to treat diseases has facilitated the identification of therapeutic agents against many diseases, such as cancer. Approximately 75% of the drugs used for cancer treatment are derived from or inspired by plants [4,5]. Various plant extracts have a strong potential to modulate apoptotic pathways [6]. Indeed, the World Health Organization (WHO) reported that various plant fractions and their active constituents are used as traditional medicines by a large part of the population [7,8].

The Bignoneaceae family is considered the second most abundant family of woody plant species in Neotropical dry forests [9] and includes 100 genera and 827 species [10,11]. In addition, indigenous cultures in South America traditionally use some of the Bignonaceae genus for different medical purposes, such as depurative, laxative, blood dysfunction, and leukemia, as well as for cultural practices, such as dyeing in ritual body paintings and protecting the skin against sunlight or repelling insects [12,13,14]. Plants of the genus *Tabebuia* (*Handroanthus*) belong to the Biognonaceae family and are Neotropical trees and shrubs that are distributed mainly in Central and South America, with relatively low contributions to African, Malagasy and Southeast Asian tropical forests [15]. There is some evidence supporting the ethnobotanical and traditional use of the genus *Tabebuia* as antimicrobial, anti-inflammatory, and anticancer agents [16,17,18], and several in vitro and in vivo studies have demonstrated its antiproliferative, anti-inflammatory, antioxidant, and antiparasitic activities [19,20,21,22,23].

*Tabebuia rosea* (Bertol.) DC, commonly called a pink trumpet, rosy trumpet tree, or guayacan, is an evergreen tree; this plant has several molecules with antiproliferative activity, such as naphthoquinones and iridoids [24,25,26]. Iridoids are naturally occurring heterocyclic monoterpenoids, such as catalposide and specioside. Several molecules with antiproliferative activity can destroy cancer cells through apoptosis, activating proapoptotic proteins such as Bax. Abnormalities in apoptotic mechanisms are hallmarks of cancer cells, and agents that activate programmed cell death could be valuable in cancer therapeutics [27]. Cell cycle control is one of the central regulatory mechanisms of cell growth. The MAPK family, which comprises serine/threonine protein kinases, is involved in the extracellular signal-regulated kinase (ERK), c-Jun-N-terminal kinase (JNK), and p38-MAPK signaling pathways; these proteins may participate in diverse cellular programs, such as the differentiation and arrest of cell cycle checkpoints, leading to apoptotic processes [28]. Indeed, p38 MAPK is a popular target in the pharmaceutical industry [29].

A promising field in current therapies based on plant-derived compounds is the study of their synergistic activity when they are administered in combination with other molecules with anticancer potential [30]. Thus, in this study, a histone deacetylase inhibitor (HDCi) was used in combination with the inner bark extract obtained from *Tabebuia rosea* as well as with two iridoids (Specioside and Catalposide) isolated previously from *T. rosea* to observe whether there was an increase in the number of apoptotic cells; apicidin (APC) is a potent HDACi that selectively binds to class I histone deacetylases (HDACs) and interferes with the deacetylation process. This process remodels chromatin and modifies the expression of genes related to apoptotic processes [31,32]. Notably, combination therapy is a strategy for overcoming resistance to anticancer drugs [33].

## 2. Results and Discussion

### 2.1. Phytochemical Analysis

A preliminary phytochemical analysis of extracts prepared from the inner bark of *T. rosea*, including the *n*-butanol extract, revealed the presence of flavonoids, lignans, terpenes, aldehydes, ketones, and unsaturated fatty acids. The results are supplied in the Supplementary Materials (Table S1).

### 2.2. Effect of the n-Butanol Extract on Cell Viability

**Figure 1:**
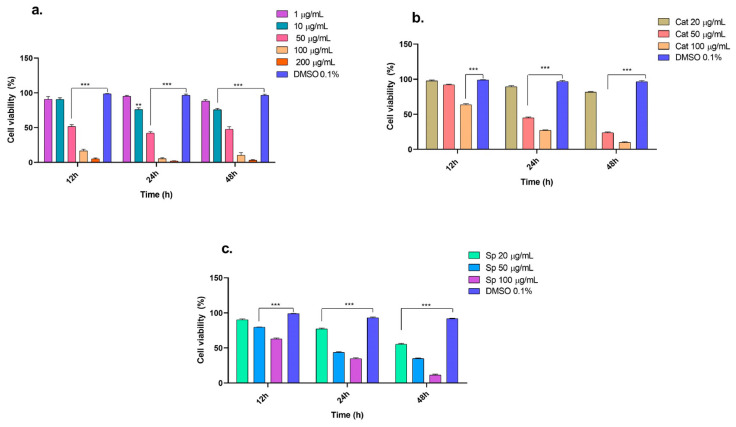
Viability of THP-1 cells in presence of (**a**) *n*-butanol extract, (**b**) catalposide, and (**c**) specioside at different incubation times (12, 24 and 48 h). ** *p* ≤ 0.01, *** *p* ≤ 0.001. ANOVA, Bonferroni post hoc correction.

Recently, drugs of a natural origin, due to their lower side effects, have been considered essential for treating several human diseases [34]. When evaluating compounds with antitumor potential, it is necessary to conduct tests to determine the selectivity of the compounds toward tumor cell lines of interest to strengthen the preclinical tests required for validation as pharmacological agents [35]. The *n*-butanol extract obtained from the inner bark of *Tabebuia rosea* and the evaluated compounds (catalposide and specioside) presented concentration-dependent cytotoxic effects on THP-1 and Jurkat cells, mainly after 24 h of treatment. Compared with that of the control cells, the viability of the THP-1 cells treated with the extracts at concentrations ranging from 10, 50, 100, and 200 μg/mL was significantly lower (*p* < 0.05) (Figure 1). Similar results were obtained with extracts from the inner bark of *Tabebuia pallida* in Ehrlich ascites carcinoma (EAC) cells, where viability was inhibited below 50% at 24 h at concentrations of 8–120 μg/mL [36]. The IC_50_ values for the *n*-butanol extract, catalposide, and specioside at 24 h were 44.7, 43.9, and 40.3 μg/mL, respectively (Table 1). These concentrations are similar to those reported by Rahman et al. for the inner bark extract of *T. pallida* [36]. According to Kuete and Effath (2015), the compounds evaluated in that study have moderate cytotoxicity since, at 24 h of exposure, the IC_50_ was in the range of 20–50 μg/mL [37].

### 2.3. Selectivity Index

In terms of the selectivity of the compounds, the *n*-butanol extract and the compounds were selective for the THP-1 cell line, mainly at 24 h, since the selectivity indices are between two and three, whereas for the Jurkat cell line, the SI was lower than one (Table 1). The differences in cytotoxicity between Jurkat and the THP-1 cells can be explained by the presence of intracellular signaling proteins, such as caveolin 1, that can affect membrane transport and the cell cycle. Some studies report an increase in the expression of this protein in cells of myeloid origin, increasing the sensitivity of these cells to molecules with antiproliferative potential [38,39]. In the literature, extracts or molecules with SI values of two or more are considered to be selective for tumor cells; this means that the evaluated compound is twice as cytotoxic for the tumor cell line as it is for normal cells [40]. Peripheral blood mononuclear cells (PBMCs) were used as normal cells. Considering the results obtained, apoptosis assays were performed in the THP-1 cell line.

### 2.4. Nuclear Morphology of Cells Treated with an n-Butanol Extract from the Inner Bark of Tabebuia rosea and Pretreated with Apicidin

**Figure 2:**
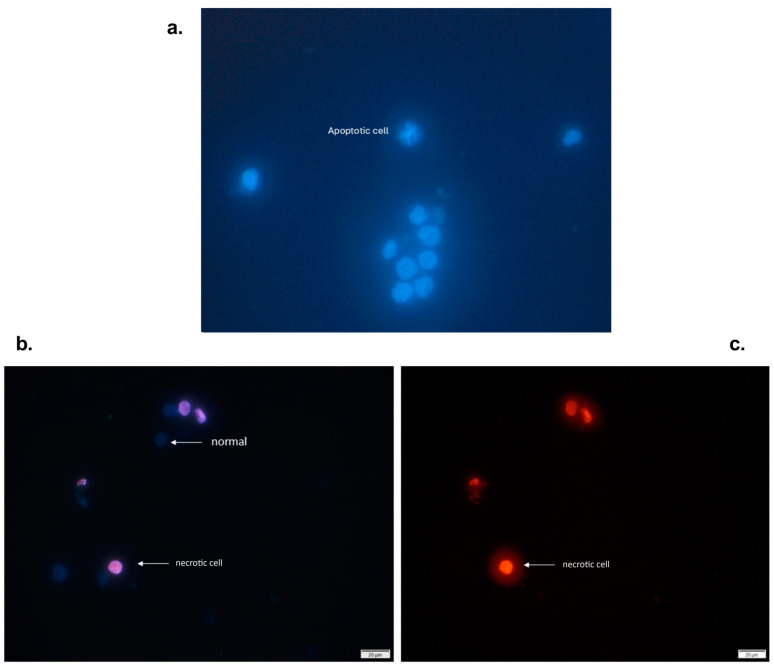
Viable and apoptotic THP-1 cells (with fragmented nuclei) were exposed to different concentrations of the *n*-butanol extract obtained from the inner bark of *T. rosea* for 24 h. (**a**) Viable (normal) THP-1 cells marked with Hoechst 33342. (**b**) THP-1 cells stained with propidium iodide were observed, and necrotic cells were detected. In contrast, viable cells are not observed because propidium iodide is not permeable (**c**).

THP-1 cells were exposed to high, medium, or low concentrations of the extract for 24 h (Figure 2). Compared with the control, the *n*-butanol extract increased the number of cells with nuclear fragmentation, suggesting that the *n*-butanol extract has an apoptotic effect on THP-1 cells. Similarly, studies with oral carcinoma cells revealed increased internucleosomal DNA fragmentation after treatment with β-lapachone and its 3-iodine derivatives isolated from *T. avellanedae* extract [28]. In this study, pretreatment with APC increased apoptosis in cells treated with the lowest concentration of the extract (25 μg/mL). HDAC inhibitors such as apicidin have been shown to modify the expression of different genes related to cell cycle regulation, growth inhibition, and apoptosis induction [41].

These results are similar to those reported by Kwon et al., who reported that APC induces nuclear fragmentation and decreases nuclear size in HL-60 cells [42]. Another study revealed that in addition to nuclear fragmentation, APC induces apoptosis through the intrinsic pathway because it increases the level of cytochrome c and the regulation of caspase 3, 7, and 9 [43]. Moreover, a change in the membrane morphology of Ishikawa cells, from a polygonal or elongated shape to a filamentous shape, was observed after 24 h of APC treatment [44]. In this study, no changes were observed in the shape of the cell membrane, and only characteristic changes in apoptotic processes, such as nuclear fragmentation, were observed.

### 2.5. Effects of Extract, Specioside, and Catalposide on Cell Apoptosis

**Figure 3:**
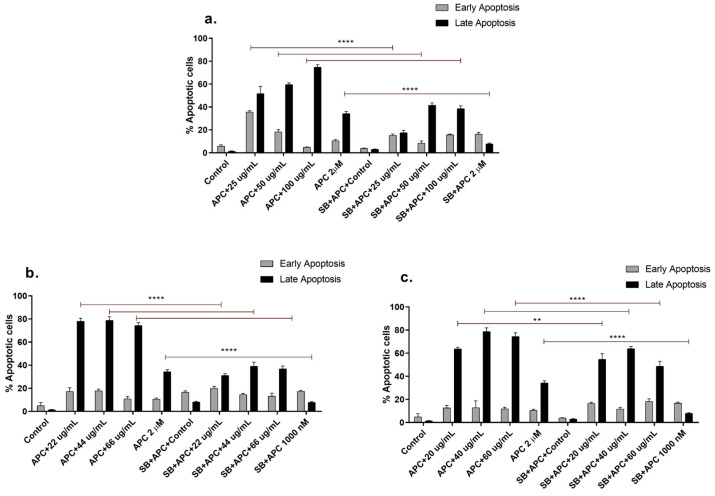
Apoptosis of THP-1 cells stained with annexin V-CF647 and 7-AAD at different concentrations of (**a**) *n*-butanol extract, (**b**) catalposide, (**c**) and specioside for 24 h and pretreated with APC and the p38 inhibitor SB202190 (SB) at 5 µM. ** *p* ≤ 0.05 **** *p* ≤ 0.0001. ANOVA, Bonferroni post hoc correction.

Apoptosis can prevent carcinogenic processes, eliminating or inhibiting the development of abnormal cells. Therefore, the induction of cancer cell apoptosis is necessary to determine the antiproliferative properties of various agents and compounds with anticancer activity [45]. The concentrations of the *n*-butanol extract evaluated in THP-1 cells at 24 h resulted in a greater percentage of early apoptotic cells. Similarly, the molecule naphthofurandione, which is present in the inner bark of *Tabebuia*, has been shown to have apoptotic effects on breast cancer cells by increasing the expression of proapoptotic proteins such as Bax [46]. Compared with catalposide or specioside treatment, apicidin pretreatment increased the percentage of apoptotic cells (Figure 3), which, at the same exposure time, increased the percentage of late apoptotic cells. Notably, only plant extracts contain bioactive molecules such as sugars, fatty acids, and antioxidants that can affect the activation of cell death pathways. In contrast, isolated compounds can directly affect late apoptosis [47].

**Figure 4:**
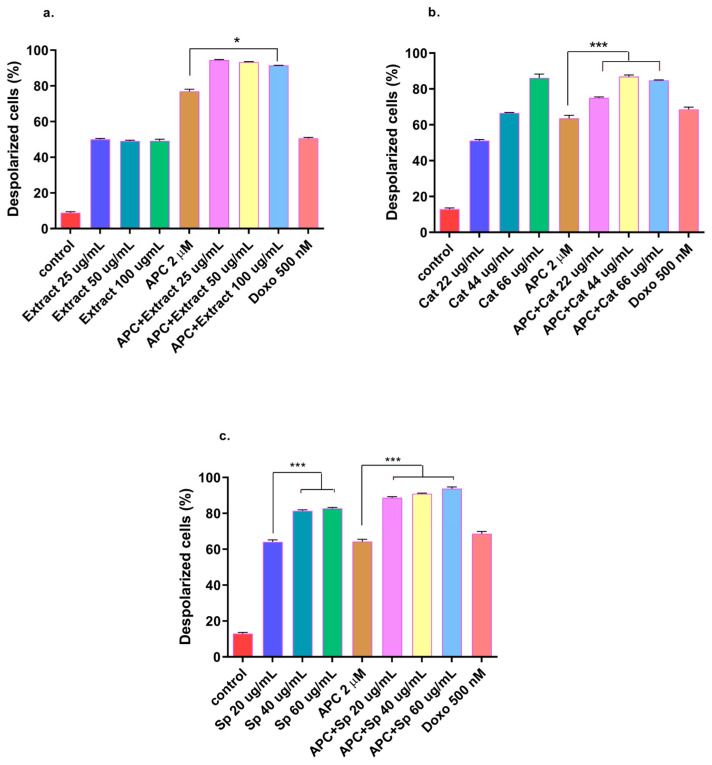
THP-1 cells with mitochondrial membrane depolarization were labeled with JC-1 and exposed to (**a**) the *n*-butanol extract obtained from the inner bark of *T. rosea* or (**b**) catalposide (Cat). (**c**) Specioside (Sp.) treatment for 24 h and pretreatment with APC for 24 h. * *p* ≤ 0.05 *** *p* ≤ 0.001. ANOVA, Tukey’s post hoc test.

**Figure 5:**
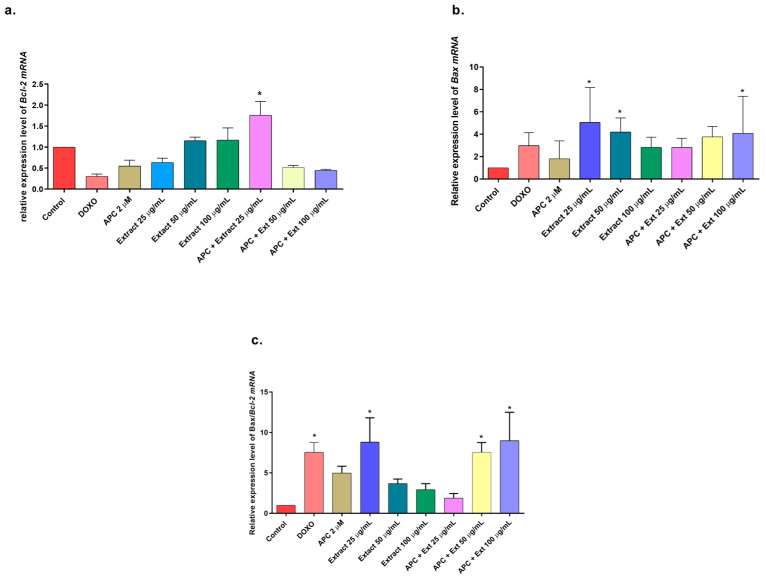
Gene expression: BCL2 and BAX genes in THP-1 cells treated with the *n*-butanol extract at different concentrations and pretreated with APC for 24 h. (**a**) BCL2 expression. (**b**) BAX expression. (**c**) Relative expression of the BAX/BCL2 genes. * *p* ≤ 0.05. Kruskal–Wallis, Dunn’s post hoc test.

**Figure 6:**
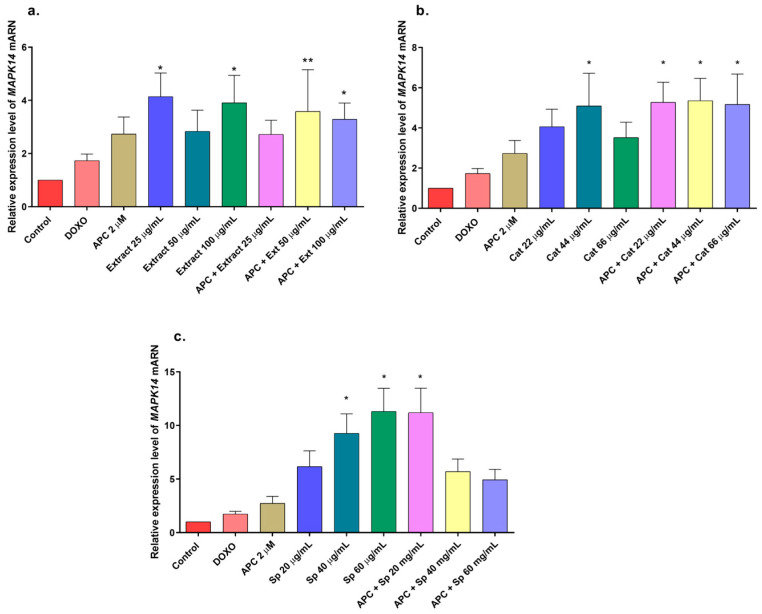
Gene expression of *MAPK14* (p38) in THP-1 cells treated with (**a**) the *n*-butanol extract and pretreated with APC for 24 h, (**b**) catalposide (Cat), and (**c**) specioside (Sp). * *p* ≤ 0.05, ** *p* ≤ 0.01. Kruskal–Wallis, Dunn’s post hoc test.

In addition to having a more significant effect on early apoptosis, the *n*-butanol extract also significantly increased (*p* < 0.05) the depolarization of the mitochondrial membrane (Figure 4) and the expression of *Bax* and *MAPK14* mRNAs at a concentration of 25 μg/mL (Figure 5 and Figure 6). However, no significant change in the protein expression of Bax or Bcl-2 was observed compared with those in the control group. In addition, *Bcl*-*2* mRNA expression was significantly decreased at several concentrations tested, except for 50 μg/mL, 100 μg/mL, and 25 μg/mL with APC pretreatment. It is essential to highlight that new transcripts of the *Bax* gene have been found in different tissues, where the encoded proteins undergo post-translational modifications that could reduce the detection of the protein with the Western blot technique [48].

In this study, compared with the control, THP-1 cells treated with catalposide or specioside presented increased apoptosis (Supplementary Materials, Figure S5). However, Saracoglu and Harpuin reported that in Hep-G2 cells, catalposide only had cytotoxic and cytostatic effects [49]. Therefore, iridoid glucosides such as catalposide may have different effects depending on the type of cancer cell.

Therefore, pretreatment with catalposide and specioside (44 and 40 μg/mL) in combination with APC induced apoptosis through the intrinsic pathway because of decreased Bcl-2 protein expression and mitochondrial membrane polarization and consequently increased expression of the Bax protein at all concentrations of the compounds and the extract used. Additionally, the conserved binding domains of Bax heterodimerize with those of Bcl-2 to decrease its antiapoptotic capacity [50]. Under normal conditions, the Bax protein is located in the cytoplasm and, in the presence of apoptotic stimuli, it migrates to the outer membrane of the mitochondria, generating the opening of the mitochondrial pores and the subsequent release of cytochrome-c into the cytoplasm [48].

**Figure 7:**
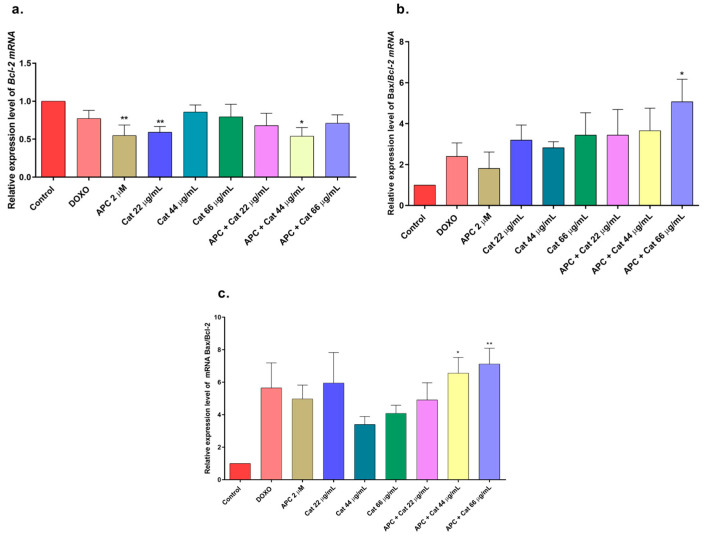
Gene expression: BCL2 and BAX in THP-1 cells treated with catalposide (Cat) and pretreated with APC for 24 h. (**a**) BCL2 expression; (**b**) BAX expression; (**c**) relative expression of the BAX/BCL2 genes. * *p* ≤ 0.05, ** *p* ≤ 0.01. Kruskal–Wallis, Dunn’s post hoc test.

**Figure 8:**
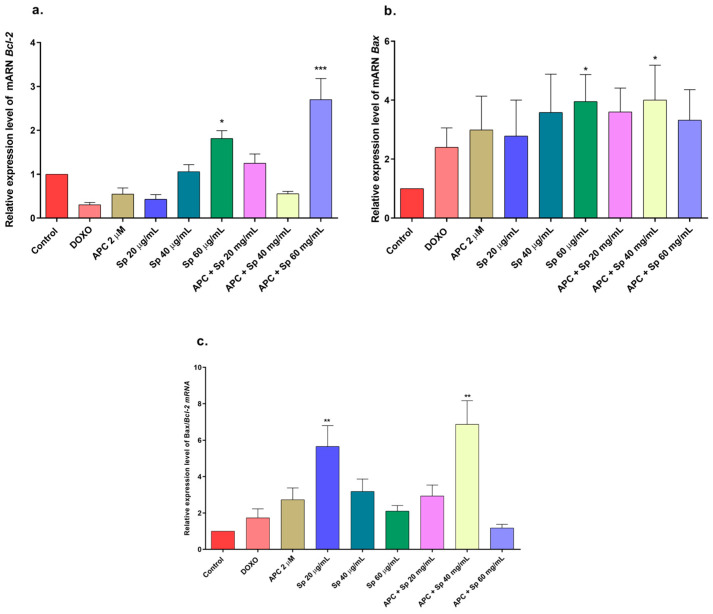
Gene expression of *BCL2* and *BAX* in THP-1 cells treated with specioside (Sp) and pretreated with APC for 24 h. (**a**) *BCL2* expression; (**b**) *BAX* expression; (**c**) relative expression of the *BAX*/*BCL2* genes. * *p* ≤ 0.05, ** *p* ≤ 0.01, *** *p* ≤ 0.001 Kruskal–Wallis, Dunn’s post hoc test.

In this study, APC decreased mitochondrial membrane polarization, and some studies reported that APC induced the accumulation of reactive oxygen species and the loss of the mitochondrial membrane potential in neuroblastoma cells [51]. Catalposide at concentrations of 44 μg/mL and 66 μg/mL, specioside at concentrations of 20 μg/mL, 40 μg/mL, and 60 μg/mL, and pretreatment with APC increased the expression of *Bax* mRNA and protein (Figure 7 and Figure 8). 

**Figure 9:**
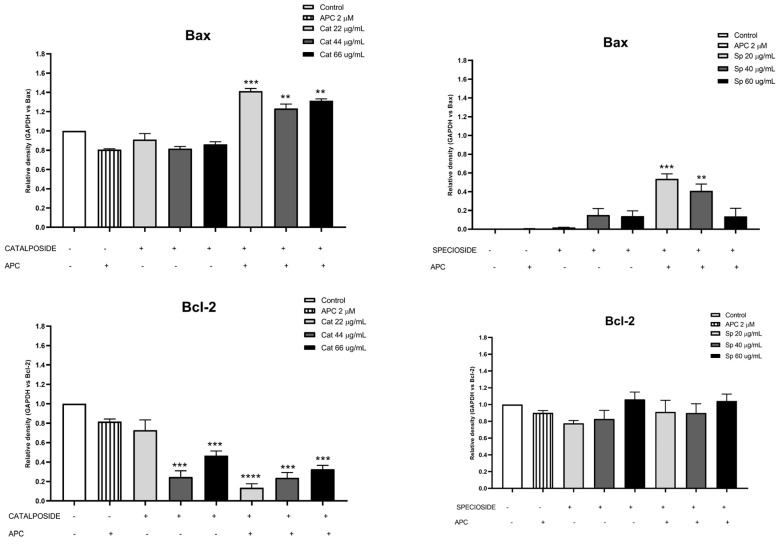
Protein expression of Bcl-2 and Bax in THP-1 cells treated with different concentrations of catalposide (Cat) and specioside (Sp) and pretreated with APC for 24 h, as determined by Western blotting. ** *p* ≤ 0.01, *** *p* ≤ 0.001, **** *p* ≤ 0.0001. Kruskal–Wallis, Dunn’s post hoc test.

When the concentration of catalposide was 44 μg/mL and that of specioside was 40 μg/mL, APC decreased the expression of *Bcl-2* mRNA. Therefore, the mean concentrations of catalposide and specioside, 44 μg/mL and 40 μg/mL, respectively, promoted apoptosis through the intrinsic pathway because of a decrease in the antiapoptotic protein Bcl-2 and an increase in the proapoptotic protein Bax (Figure 9). Additionally, a decrease in mitochondrial membrane polarization was observed at all concentrations of the compounds and the extract. The conserved binding domains of Bax heterodimerize with those of Bcl-2 to decrease its antiapoptotic capacity [50]. Under normal conditions, the Bax protein is located in the cytoplasm and, in the presence of apoptotic stimuli, migrates to the outer membrane of the mitochondria, generating the opening of the mitochondrial pores and the subsequent release of cytochrome c into the cytoplasm [48].

Some studies have reported the antiproliferative effects of the inner bark of *Tabebuia avellanedae* in MCF-7AROM cells and the extract of *Tabebuia pallida* leaves in the cancerous cells of Erlich’s ascites, which resulted in the overexpression of the *BAX* gene and decreased the expression of *BCL2* [36,46]. The evaluation of the apoptotic effect of the inner bark extract of *T. avellanedae* and some isolated compounds, such as lapacol and furanonaptoquinones, revealed that the intrinsic pathway mediates the apoptotic effect in A549 cells and canine osteosarcoma cells and that the proapoptotic proteins Bax, p53, and caspase 3 are activated [52,53]. These data demonstrate that various *Tabebuia* extracts have an apoptotic effect on cancer cells through the intrinsic pathway. Some iridoids, such as geniposidic acid, are the main compounds of *Genipa americana*; they have an apoptotic effect on synoviocytes through the intrinsic pathway, overexpressing the Bax protein and decreasing the Bcl-2 protein [54].

**Figure 10:**
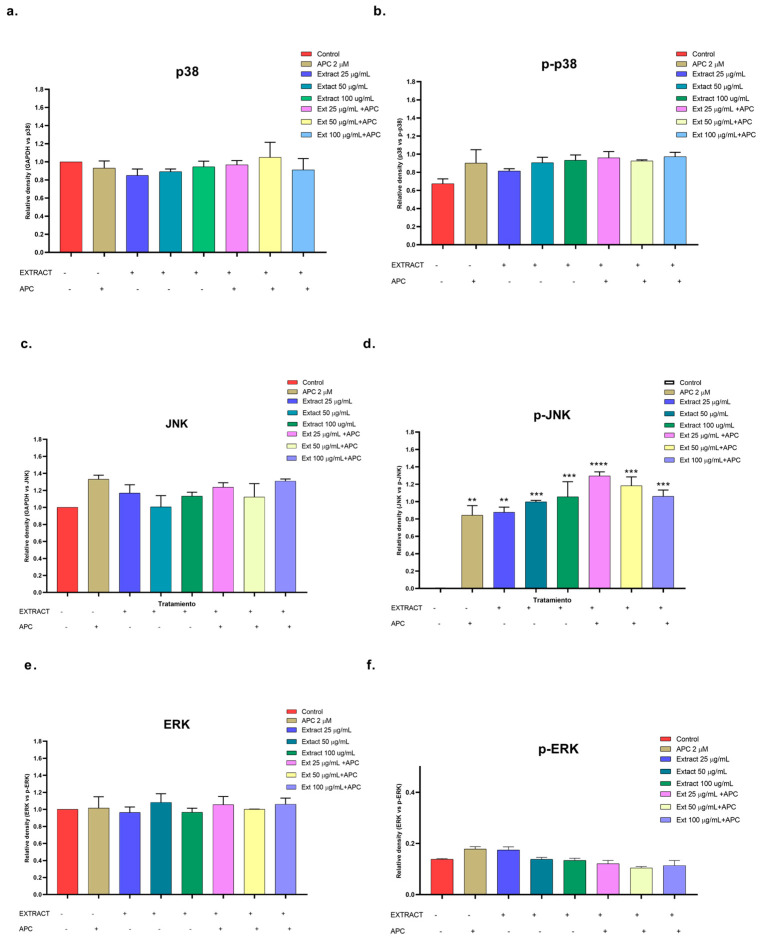
Relative protein expression density of (**a**) p38 protein, (**b**) p-p38, (**c**) JNK, (**d**) p-JNK, (**e**) ERK, (**f**) p-ERK in their basal and phosphorylated forms in THP-1 cells treated with the *n*-butanol extract and pretreated with APC for 24 h. ** *p* ≤ 0.01, *** *p* ≤ 0.001, **** *p* ≤ 0.0001. Kruskal–Wallis, Tukey post hoc test.

In this study, the *n*-butanol extract increased the mRNA expression of the *MAPK14* gene, which encodes p38, in THP-1 cells, mainly at concentrations of 25 and 100 μg/mL and in combination with APC at 50 μg/mL (Figure 6); a slight increase in the phosphorylation of the p38 protein was observed at all the concentrations evaluated compared with that in the control (Figure 10a,b). The JNK protein was significantly phosphorylated at all the concentrations of the *n*-butanol extract evaluated; pretreatment with APC and 25 μg/mL had the greatest effect on the level of the protein (Figure 10c,d), whereas catalposide did not significantly change the level of JNK compared with that of the control. Specioside significantly phosphorylated the JNK protein at all concentrations tested, except at 60 μg/mL, in the presence of APC. Compared with the control, the *n*-butanol extract and specioside did not significantly change the phosphorylation of the ERK protein (Figure 10e,f). In contrast, compared with the control, catalposide decreased protein phosphorylation but not significantly. Some secoiridoids, such as oleuropein, have been shown to induce apoptosis through the JNK pathway in A549 cells and decrease the mitochondrial membrane potential [55,56].

**Figure 11:**
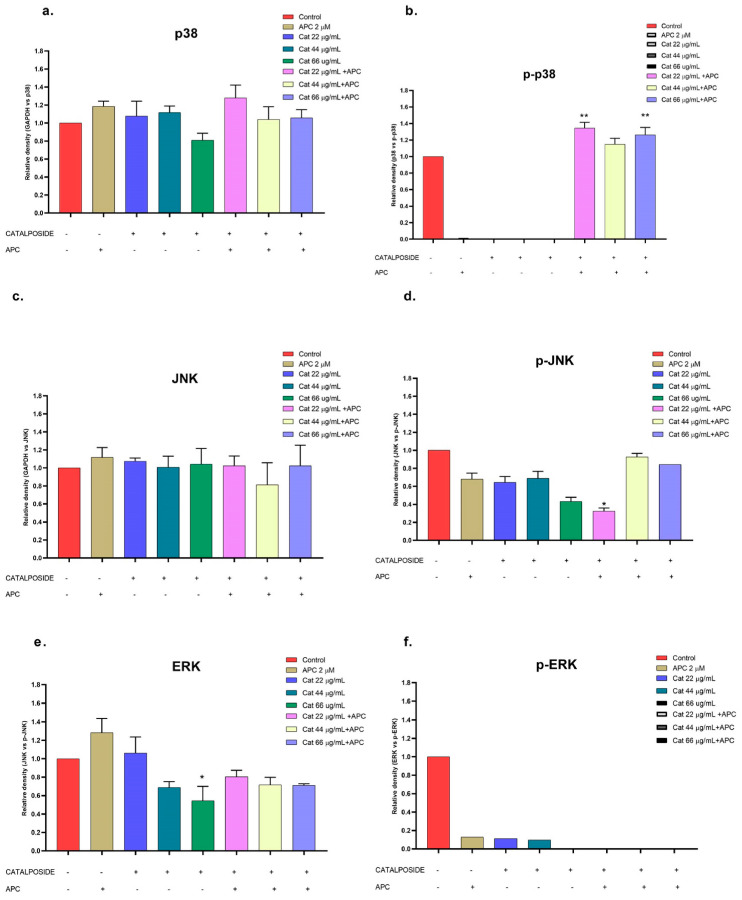
Relative protein expression density ((**a**) p38 protein, (**b**) p-p38, (**c**) JNK, (**d**) p-JNK, (**e**) ERK, (**f**) p-ERK) in their basal and phosphorylated forms in THP-1 cells treated with catalposide (Cat) and pretreated with APC for 24 h. * *p* ≤ 0.05, ** *p* ≤ 0.01. Kruskal–Wallis, Dunn’s post hoc test.

**Figure 12:**
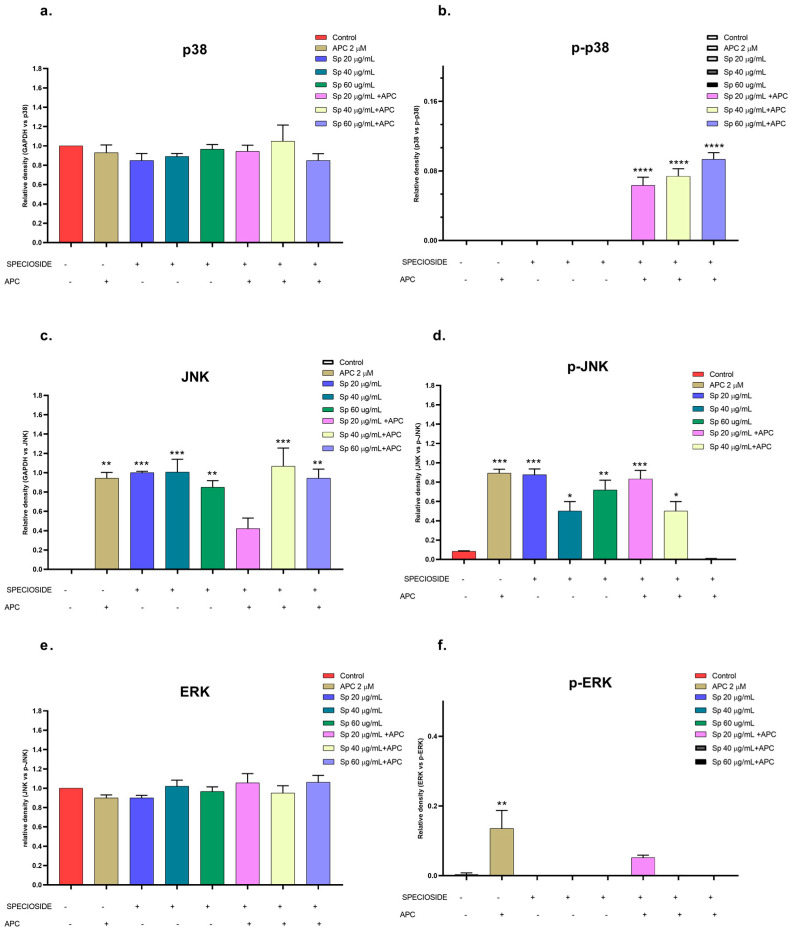
Relative protein expression density (**a**). p38 protein, (**b**). p-p38, (**c**). JNK, (**d**). p-JNK, (**e**). ERK, (**f**). p-ERK) in their basal and phosphorylated forms in THP-1 cells treated with specioside (Sp) and pretreated with APC for 24 h. * *p* ≤ 0.05, ** *p* ≤ 0.01, *** *p* ≤ 0.001, **** *p* ≤ 0.0001. Kruskal–Wallis, Dunn’s post hoc test.

Catalposide and specioside significantly increased the phosphorylation of p38 in the cells pretreated with APC compared with the control (*p* < 0.001) (Figure 11 and Figure 12). These data are related to the increase in the expression of *MAPK14* mRNA. It has been reported that the phosphorylation of MAPK kinases such as p38 can be inhibited by some molecules that are present in plant extracts, such as flavonoids that inhibit the kinases MKK3 and MKK6, proteins that participate in the phosphorylation of the conserved Thr-Gly-Tyr (TGY) motif of p38 [57].

Cell cycle alteration is an essential feature in the transformation of normal cells into tumor cells and, therefore, has therapeutic potential for several regulatory molecules because it controls cell proliferation. The cell cycle distribution was analyzed to identify the mechanism of action of the *n*-butanol extract, and the compounds were evaluated for their ability to decrease the activity of THP-1 cells. The results with the *n*-butanol extract revealed a slight increase in the percentage of cells in the G0/G1 phase (Supplementary Materials, Figure S6a), whereas for catalposide and specioside, the increase was significant compared with that of the control (Supplementary Materials, Figure S6b,c). In addition, APC pretreatment increased G0/G1 phase arrest. The cell cycle regulates cell growth and proliferation; when it is arrested, it can inhibit proliferation and induce apoptosis in cancer cells [58]. In this study, compared with control cells, cells treated with catalposide, specioside, or APC presented significant phosphorylation of the p38 protein; these findings suggest that this protein is involved in cell cycle arrest in the G0/G1 phase. However, identifying the activity of other proteins, such as cyclin D1, related to this cell cycle phase is necessary. In one study, the extract obtained from *Galium aparine*, a plant that contains large amounts of iridoids, arrested the cell cycle at 72 h in G0/G1 and increased the fraction of cells in the subG1 phase, generating an apoptotic effect in MCF-7 cells [59,60]. It has also been reported that some flavonoids, such as apigenin and monoterpenes (1,8 cineol), are able to arrest the cell cycle in the G0/G1 phase and phosphorylate the p38 protein after 24 h of exposure. Additionally, they induce the expression of the p21 protein, which acts upstream of the p38 MAPK pathway [61,62].

The findings of this study highlight the potential of natural products in the search for plant extracts and new molecules with antineoplastic activity. The *n*-butanol extract obtained from the inner bark of *Tabebuia rosea* (Bertol.) DC and the iridoids catalposide and specioside, in the presence of apicidin, induces apoptosis in leukemia cells. This work also has ethnopharmacological importance, since several indigenous groups in Central and South America use *Tabebuia* bark as an herbal or medicinal tea to treat diseases such as cancer and infectious diseases [23,63,64]. However, the cell signaling pathways involved in the antiproliferative and anticancer activities of *T. rosea* inner bark need further study.

## 3. Materials and Methods

### 3.1. Plant Material and Preparation of Extract

The inner bark of *T. rosea* (Bertol.) DC was collected at the Universidad Tecnológica de Pereira Campus. The plants were identified at the Colombian National Herbarium (Voucher No. COL 582577 for *T. rosea*). The collection and processing of the material were covered by collection permission number 1133/2014, which was issued by the Autoridad Nacional de Licencias Ambientales (ANLA) de Colombia.

All the chemicals used in this study were of analytical grade. The extract was obtained by the Polifenoles Research Group, as described by Jimenez et al. in 2018 [23]. Briefly, the plant material from the inner bark of *T. rosea* (2 kg) was dried and macerated in analytical-grade methanol (14 L) for 48 h. To obtain the crude extract, it was necessary to homogenize, filter, and concentrate it under vacuum using a rotary evaporator (Heidolph, Laborota model, Wood Dale, IL, USA) at 40 °C. This procedure was repeated three times. The crude extract was dissolved in 400 mL of distilled water and subjected to liquid—liquid extraction with n-butanol; the extract was vacuum dried by a vacuum rotary evaporator, and a mass of 12.5 g was obtained. Endotoxin levels in the extract were assayed using the Limulus Amebocyte Lysate Test, E-Toxate Kit (Sigma Chemical Co., Saint Louis, MO, USA; Cat No. ET0200-1KT). The sample was negative for the presence of endotoxins (detection limit 0.05–0.1 EU/mL). The extract was refrigerated at −20 °C in an amber tube protected from light, heat, air, and moisture. For each of the biological assays, the extracts were dissolved in DMSO (dimethyl sulfoxide, Merck, Darmstadt, Germany; Cat No. 1029521000).

### 3.2. Preliminary Phytochemical Analysis

Preliminary phytochemical analysis was performed by selective derivatization reactions to characterize the secondary metabolites present in the *n*-butanol extract obtained from the inner bark of *T. rosea*. The characterization was carried out using thin-layer chromatography (TLC) in the normal phase (silica) and reversed-phase (RP-18) with hexane-ethyl acetate (7:3) and water-isopropanol (7:3) elution systems, respectively. The chromatographic plates were developed with aluminum chloride (AlCl_3_, Sigma Chemical Co., Saint Louis, MO, USA), ferric chloride (FeCl_3_, Sigma Chemical Co., Saint Louis, MO, USA), and a mixture of vanillin-phosphoric acid (H_3_PO_4_) for the detection of flavonoids, phenols, and lignans. Potassium hydroxide (KOH, Merck, Darmstadt, Germany) in analytical-grade ethanol was used for the detection of anthrones, quinones, and coumarins; a mixture of anisaldehyde–acetic acid–sulfuric acid (H_2_SO_4_) with vanillin-phosphoric acid (H_3_PO_4_) was used for the detection of iridoids; a mixture of anisaldehyde–acetic acid–sulfuric acid (H_2_SO_4_) with antimony chloride (SbCl_3_) was used for the detection of saponins and triterpenes; oleum (Sigma Chemical Co., Saint Louis, MO, USA) was used for the detection of sesquiterpene lactones; 2,4-dinitrophenylhydrazine was used for the determination of aldehydes and ketones; and the Liebermann–Burchard reagent was used for the detection of terpenes and steroids. The extract showed a specific color when reacted with the developing reagents for each test. The absence or presence of this color was taken as a negative (−) or positive (+) result for the presence of these phytochemical components.

### 3.3. Fractionation and Isolation of Specioside

The isolation and structural identification of specioside (the principal constituent of the *n*-butanol extract from *T. rosea*) was previously reported [22]. The *n*-butanol extract was concentrated via rotary evaporation under reduced pressure, and a dark brown extract (12.5 g, yield 4.25%) was obtained. The *n*-butanol extract (8.0 g) was subjected to separation by column chromatography (CC) on a Diaion^®^ HP-20 (Mitsubishi Chemical Corp., Tokyo, Japan), with a water—isopropanol elution gradient (90:10 to 10:90), obtaining subfractions A–J. Tr-1 (Specioside, 25.9 mg) was isolated from subfraction D with a semipreparative HPLC–DAD system (Hitachi-Merck, Tokyo, Japan) in reverse phase (LiChrocart 250-10; LiChrospher 100; 10 µm, Merck, Darmstadt, Germany) by isocratic elution with H_2_O–ACN (70:30% *v*/*v*) containing 1% *v*/*v* CH_3_COOH. Specioside (Figure S7) was obtained as a dark brown amorphous solid (m.p. 142–162 °C). The FTIR spectrum displayed absorption bands attributable to carbonyl groups (ν_C=O_ 1698 cm^−1^), hydroxyl groups (ν_O-H_ 3383 cm^−1^), and aromatic rings (ν_C=C_ 1605 cm^−1^).

Full assignments from the ^1^H and ^13^C NMR spectra were made through the use of ^1^H-^1^H COSY, HSQC, and HMBC experiments. All the experiments were performed on a 400 MHz Agilent spectrometer (Agilent, Santa Clara, CA, USA) using deuterated methanol as the solvent. The ^1^H NMR spectrum showed two olefinic protons at δ H 6.37 (H-3, dd) and δ H 4.98 (H-4, dd), characteristic of the iridoid nucleus. This structure was confirmed by correlations shown in the HMBC spectrum with carbons at δ C 140.95 (C-3) and δ C 101.50 (C-4). In addition, two olefinic protons at δ H 7.67 (1H, d, *J* = 16.0 Hz, H-7″) and δ H 6.38 (1H, d, *J* = 15.9 Hz, H-8″) suggested the presence of an E configuration, which is characteristic of a p-coumaroyl skeleton. The p-coumaroyl structure was confirmed by the observation of two signals at δ H 7.48 (2H, d, *J* = 8.7 Hz, H-2″, H-6″) and δ H 6.81 (2H, d, *J* = 8.7 Hz, H-3″, H-5″), characteristic of an AA′XX′ system; these data were confirmed by the ^13^C NMR spectrum, which exhibited eight carbon signals, including the carbonyl carbon at δ C 164.49 (C-9″), which was attributed to the p-coumaroyl ester. The presence of anomeric protons at δ H 4.79 (1H, d, *J* = 7.9 Hz, H-1′) and methine signals at δ H 3.42–3.23 (4H, m) are characteristic of a sugar moiety. Analysis of the 1D and 2D NMR spectra, in addition to comparisons with the literature data for glucoside analogs, suggested that the saccharide portion was a glucose moiety. Characteristic ^1^H NMR, ^13^C NMR, COSY, HSQC, and HMBC spectra are provided in the Supplementary Materials (Figures S7–S13).

The molecule catalposide, an iridoid glycoside also isolated from the plants of the genus *Tabebuia* [65], was purchased from Sigma Chemical Company (SMB00094).

### 3.4. Cell Culture

Jurkat and THP-1 cell lines were purchased from the American Type Culture Collection (ATCC, Rockville, MD, USA); Jurkat is a human lymphocytic cell line representing acute T-cell leukemia, and THP-1 is a monocytic cell line that represents acute myelogenous leukemia (AML). Jurkat and THP-1 cells were maintained in RPMI 1640 medium (Sigma) supplemented with 10% heat-inactivated fetal bovine serum (FBS) (Gibco Cat No. 16140071, Waltham, MA USA), 200 μg/mL penicillin, 200 μg/mL streptomycin, 400 μg/mL neomycin (GIBCO, Cat No. 15640-055), 5 μg/mL amphotericin, and 1 mM sodium pyruvate (all from Sigma Chemical Co., Saint Louis, MO, USA; Cat Nos. A9528-50 MG, M7522-100ML, and S8636-100ML, respectively). The cells were maintained at 37 °C in a humidified atmosphere with 5% CO_2_. The culture medium was exchanged with fresh medium every 2–3 days. Peripheral blood mononuclear cells (PBMCs) were isolated from normal human venous blood. Further, 10 milliliters of human blood was mixed with 10 mL of RPMI-1640 containing 50 µL of heparin and gradually poured over 1 mL of HISTOPAQUE^®^-1119 (Sigma). The plasma was removed by centrifugation at 3000 rpm for 30 min. The peripheral blood mononuclear cell-containing layer was collected with a pipette and diluted in 4 mL of PBS. PBMCs were harvested by centrifugation at 1500 rpm for 10 min at room temperature, resuspended in RPMI-1640 with 10% FBS, and incubated at 37 °C in 5% CO_2_ humidified air.

### 3.5. Cell Viability Assay (MTT)

Cell viability was determined by measuring the mitochondrial activity of live cells via the metabolism of the tetrazolium substrate 3-(4,5-dimethylthiazol-2-yl)-2,5-diphenyl tetrazolium bromide (MTT) and converting it to purple formazan crystals [66]. Briefly, the cells (2 × 10^4^ cells/well) were treated with 25, 50, 100, 200, or 400 μg/mL extract diluted in DMSO (at a final concentration of 0.5%) and incubated for 12, 24, or 48 h. Catalposide and specioside were tested at concentrations of 20, 50, and 100 μg/mL for 12, 24, and 48 h. For apicidin, 0.5, 1, and 2 μM apicidin were tested for 24 and 48 h. Doxorubicin was used as a reference standard drug (positive control). After treatment, the medium was discarded; 200 μL of RPMI 1640 medium without phenol red containing 0.5 mg/mL MTT (Sigma Chemical Co., Saint Louis, MO, USA; Cat. No. M2128-500MG) was then added to each well. The plates were incubated for four hours at 37 °C with 5% CO_2_ in a humidified atmosphere. The medium was discarded, and 100 μL of DMSO was added to solubilize the formazan crystals. The absorbance was measured with an ELISA microplate reader at 492 nm (ELx800; BioTek Instruments Inc., Winooski, VT, USA). The percentage of viable cells was calculated on the basis of the nontreated control (taken as 100%). The IC_50_ (concentration causing a 50% reduction in cellular viability) values for all the tested compounds were evaluated in Excel using the nonlinear regression curve method. Three independent assays were performed, each in triplicate.

### 3.6. Estimation of the Selectivity Index (SI)

The selectivity index corresponds to the ratio of the IC_50_ value calculated for the activity of synthesized compounds on a regular cell line (PBMCs) to that of the IC_50_ calculated for cancer cell lines (Jurkat and THP-1) [67]. Values greater than two were optimal for an efficient selectivity index.

Selectivity index:

(1) $$SI=\frac{{\mathrm{IC}}_{50}\;\mathrm{normal}\;\mathrm{cells}}{{\mathrm{IC}}_{50}\;\mathrm{cancer}\;\mathrm{cells}}$$

### 3.7. Fluorescence Microscopic Analysis of Cell Death (Propidium Iodide and Hoechst Staining)

Fluorescence microscopy was used to observe cell nuclear morphology, followed by propidium iodide (50 µg/mL) and Hoechst 333342 (1 mg/mL) staining. THP-1 cells (1 × 10^5^ cells/well) were treated with fresh media (control) and with 25, 50, or 100 µg/mL extract for 24 h; 400 µM doxorubicin was used as a positive control. Further, 10 microliters of the cell suspension was mixed with 90 µL of the dye, incubated in a water bath for 5 min at 37 °C, and wrapped in aluminum foil. The cells were examined under an Olympus IX53 fluorescence microscope (Olympus Corporation, Tokyo, Japan). The following formula was used to calculate the percentage of apoptotic cells [68].

### 3.8. Flow Cytometric Analysis of Apoptosis via the ANNEXIN V-CF647/7-AAD Double-Staining Assay

In 96-well plates, 8 × 10^4^ cells were treated with 25, 50, and 100 µg/mL extract and 400 µM doxorubicin (positive control) for 24 and 48 h. The cells were washed with 1X PBS, and apoptotic cells were detected by staining with 100 µL of Annexin V-CF647 and 5 µL of the 7-AAD Apoptosis Annexin Red Kit (FCCH100108 FlowCellect, Millipore, Darmstadt, Germany). The cells were incubated at room temperature for 15 min in the dark and analyzed on a Guava Millipore flow cytometer (Merck Millipore, Bedford, MA, USA) using InCyte software (version 3.1, Merck Millipore). The percentage of annexin V-positive cells was evaluated.

### 3.9. Determination of Mitochondrial Membrane Potential (ΔΨm)

The Λᴪm was measured using a Guava Mitochondrial Depolarization Kit (4500-0250 Millipore, Darmstadt, Germany). After pretreatment with 1 µM apicidin for 24 h, the cells were exposed to various concentrations of the extract (25, 50, and 100 µg/mL) for 24 h. The cells were collected and suspended in 100 µL of PBS containing JC-1 and 7-AAD after incubation at 37 °C for 15 min. The fluorescence intensity of the cells was analyzed in a Guava Millipore flow cytometer (Merck Millipore, Bedford, MA, USA) using InCyte software (version 3.1, Merck Millipore).

### 3.10. Cell Cycle Analysis

After treatment with the extract, 7 × 10^4^ cells were harvested, washed with PBS, fixed with 200 µL of ice-cold 70% ethanol, and maintained at 4 °C for 4 h before staining. The cell pellet was washed in 1X PBS, resuspended in 200 µL of Guava Cell Cycle reagent (#4500-0220, Millipore), and incubated in the dark at 37 °C for 30 min before analysis with a Guava flow cytometer (Merck Millipore, Bedford, MA, USA) using InCyte software (version 3.1, Merck Millipore).. Centrifugation was performed at 1200 rpm for 5 min at room temperature for each step.

### 3.11. Western Blot Analysis

THP-1 cells were seeded in 24-well plates (5 × 10^5^ cells/well), and after 24 h of incubation, they were pretreated with 1 µM apicidin for 24 h and then treated with different concentrations of the *n*-butanol extract, catalposide, and specioside for 24 h. After treatment, the cells were lysed with an Abcam protein extraction kit (ab270054), and a phosphatase inhibitor cocktail (ab201115) was added. The supernatants of the cell extracts were quantified with a BCA protein assay (Thermo Scientific Pierce #23227, Waltham, MA, USA). Equivalent amounts of protein were electrophoresed on 10% SDS polyacrylamide gels and then electroblotted onto 0.45 μm polyvinylidene difluoride (PVDF) membranes (GE Healthcare, Chicago, IL, USA). After blocking with 5% nonfat milk in TBS/0.5% Tween 20 (TBST) for 1 h at room temperature, the membranes were incubated with p-p38 MAPK (1:1000 dilution, cat 701057), p38 MAPK (1:2000 dilution, cat PA5-17713), p-ERK1/2 (1:100 dilution, 44680G), ERK1/2 (1:2000 dilution, 44654G), p-JNK1/2 (1:1000 dilution, PA5-37698), JNK1/2 (1:2000 dilution, cat 44690G), bcl-2 (1:2000 dilution, PA1-28275), Bax (1:2000 dilution, PA5-70418), and GAPDH (1:10,000 dilution, PA1-16777) antibodies overnight at 4 °C. All the antibodies were purchased from Invitrogen-Thermo Fisher Scientific (Waltham, MA, USA). Afterward, the membranes were washed with TBST three times and incubated with horseradish peroxidase (HRP)-conjugated secondary antibody for 1 h at room temperature. The signals were detected using SuperSignal™ West Pico PLUS Chemiluminescent Substrate (Thermo Scientific Pierce cat 34577) and analyzed with a ChemiDoc XRS+ system (Bio-Rad, Hercules, CA, USA). The density of each band was measured using ImageJ software V. 1.53n, 7 November 2021 (NIH, Bethesda, MD, USA), and the protein levels were normalized to the GAPDH levels. The data are expressed as the means of three independent experiments. The Western blot images are presented in the Supplementary Materials (Figures S1–S4).

### 3.12. RT–qPCR

THP-1 cells (3 × 10^5^ cells/well) were pretreated with 1 µM apicidin for 24 h and then treated with different concentrations of the extract, catalposide, and specioside (25, 50, 100 µg/mL) for 24 h. After treatment, mRNA extraction was performed using the RNeasy Plus Mini Kit (Qiagen, Germantown, MD, USA; Cat No. 74134). The mRNA was quantified with a NanoDrop 2000c (Thermo Fisher Scientific, Waltham, MA, USA). The expression of the *MAPK14* (gene encoding p38), *BCL2*, and *BAX* genes was evaluated by qRT–PCR and quantified with the 2^−ΔΔCt^ method in the Applied Biosystems StepOnePlus Real-Time PCR System (Applied Biosystems, Foster City, CA, USA), which uses predesigned TaqMan Gene Expression Assays (codes Hs00608023_m1, Hs00608023_m1, and Hs00180269_m1, respectively) (Applied Biosystems, Foster City, CA, USA) and the TaqMan^®^ RNA-to-CT TM 1-Step Kit (Applied Biosystems, Foster City, CA, USA, Cat No. 4392653). The reaction mixture was incubated at 48 °C for 15 min and 95 °C for 10 min and then cycled (40 cycles) at 95 °C for 15 s and 60 °C for 1 min. GAPDH was used as an endogenous control.

### 3.13. Statistical Analysis

Each experiment was performed at least in duplicate. The results are expressed as the means ± SDs of at least three independent experiments. Statistical analysis was performed using ANOVA and the Kruskal–Wallis test. Values were considered significantly different at *p* < 0.05. The statistical tests were performed using GraphPad Prism, version 8.01 (GraphPad Software, San Diego, CA, USA).

## 4. Conclusions

Pretreatment with APC, the *n*-butanol extract obtained from the inner bark of *Tabebuia rosea*, and the evaluated compounds (catalposide and specioside) activated the MAPK pathway, specifically p38, which affected the G0/G1 phase of the cell cycle and subsequent cell death; although the JNK and ERK proteins were expressed in the cells treated with the catalposide and specioside compounds, the phosphorylation of these proteins was not significant. In addition, there was depolarization of the mitochondrial membrane, an effect that was related to the participation of the proapoptotic protein Bax.

## Figures and Tables

**Table 1:** IC_50_ (µg/mL) and SI of the *n*-butanol extract of *T. rosea*, catalposide, specioside, and doxorubicin in THP-1 and Jurkat cells (median ± SD, *n* = 3).

Time	12 h			24 h			48 h		
Cell Line	Jurkat	THP-1	PBMC	Jurkat	THP-1	PBMC	Jurkat	THP-1	PBMC
**Extract**	314.8 ± 2.2	35.3 ± 3.2	340.5 ± 2.22	189.5 ± 0.5	44.7 ± 4.082	123.64 ± 5.2	354.3 ± 0.1	36.44 ± 1.4	99.4 ± 4.7
**SI**	1.08	**9.64**		0.65	**2.77**		0.28	**2.72**	
**Catalposide**	106.74 ± 5.3	121.6 ± 3.3	125.67 ± 2.8	92.77 ± 1.5	43.86 ± 2.2	87.7 ± 3.1	71.16 ± 2.8	30.16 ± 1.67	43.4 ± 3.4
**SI**	1.18	1.03		0.94	1.99		0.61	1.44	
**Specioside**	137.66 ± 1.3	47.25 ± 2.3	165.25 ± 3.1	81.71 ± 3.5	40.32 ± 1.3	70.66 ± 3.7	68.86 ± 2.1	26.33 ± 6.8	54.22 ± 3.7
**SI**	1.20	**3.49**		0.86	1.75		0.79	**2.06**	
**Doxorubicin (µg/mL)**	0.339 ± 1.2	1.268 ± 0.2	1.364 ± 0.15	0.258 ± 0.1	0.189 ± 0.1	0.224 ± 0.1	0.050 ± 0.0	0.005 ± 0.01	0.018 ± 0.1
**SI**	**4.02**	1.07		0.86	1.183		0.36	**3.6**	

## Data Availability

The original contributions presented in the study are included in the article, and further inquiries can be directed to the corresponding author.

## Supplementary Materials

The following supporting information can be downloaded at https://www.mdpi.com/article/10.3390/molecules29173986/s1, Supplementary information Guerrero-Pepinosa et al. Table S1. Preliminary phytochemical analysis of extracts prepared from the inner bark of *T. rosea*. Figure S1. Blotting of MAPK proteins (p38 protein, p.p38, JNK, p-JNK, ERK, p-ERK). in their basal and phosphorylated forms in THP-1 cells treated with the n-butanol extract and pretreated with APC for 24 h. Figure S2. Protein expression of Bcl-2 and Bax in THP-1 cells treated with different concentrations of catalposide (Cat) (a) and specioside (Sp) (b) and pretreated with APC for 24 h, as determined by western blotting. Figure S3. Relative protein expression density (p38 protein, p-p38, JNK, p-JNK, ERK, p-ERK). in their basal and phosphorylated forms in THP-1 cells treated with catalposide (Cat) and pretreated with APC for 24 h. Figure S4. Relative protein expression density (p38 protein, p-p38, JNK, p-JNK, ERK, p-ERK). in their basal and phosphorylated forms in THP-1 cells treated with specioside (Sp) and pretreated with APC for 24 h. Figure S5. THP-1 cells labeled with Annexin V-CF647 and 7-AAD in early and late apoptosis and treated with different concentrations of Catalposide (a) and Specioside (b) for 24 hours. ** *p* ≤ 0.01,**** *p* ≤ 0.0001. ANOVA, Bonferroni post hoc. Figure S6. (a) THP-1 cells in each phase of the cell cycle were exposed to the n-butanol extract obtained from the inner bark of *T. rosea*. (b) Pretreatment with the catalposide (Cat) and (c) specioside (Sp) and pretreatment with APC for 24 h. * *p* ≤ 0.05, *** *p* ≤ 0.001. Kruskal–Wallis, Dunn’s post hoc test. Figure S7. Specioside structure. Figure S8. The ^1^H NMR spectrum of Specioside in CD3OD, 400 MHz. Figure S9. The ^13^C NMR spectrum of Specioside in CD3OD, 125.6 MHz. Figure S10. The COSY spectrum of Specioside in CD3OD, 400 MHz. Figure S11. The HSQC spectrum of Specioside in CD3OD, 400 MHz. Figure S12. The HMBC spectrum of Specioside in CD3OD, 400 MHz. Figure S13. The HMBC expanded spectrum of Specioside (δ 6.4–3.2). Figure S14. The HMBC expanded spectrum of Specioside (δ 7.8–6.2).
