# Supplementary material for: The n-Butanol Extract Obtained from the Inner Bark of Tabebuia rosea (Bertol.) DC, Specioside, and Catalposide Induce Leukemia Cell Apoptosis in the Presence of Apicidin
Source: Molecules. 2024 Aug 23;29(17):3986. doi: 10.3390/molecules29173986 (PMC11396062; doi:10.3390/molecules29173986)

Supplementary material Guerrero-Pepinosa et al.

Table S1. Preliminary phytochemical analysis of extracts prepared from the inner bark of *T. rosea*

PHYTOCHEMICAL COMPONENT	REAGENT	MEOH	HEX	$CHCL_3$	ACET (SUN)	ACET (INS)	BUOH	H_2O
**PHENOLS AND TANNINS**	$FeCL_3$/EtOH	+	-	++	+	+	+	-
**FLAVONOIDS**	$AlCl_3$/EtOH	+++	+	+	+++	+++	+++	+
**LIGNANS**	UV 365 nm	++	-	+	++	++	++	+
	Vanillin/EtOH-H_3PO_4	+++	+++	+++	+++	+++	+++	++
**ANTHRONES**	KOH/EtOH	++	-	-	-	++	++	++
**ANTHRAQUINONES**		+	-	+++	-	-	-	-
**COUMARINS**		++	++	+++	+++	+++	+++	+
**TERPENES/TERPENOIDS STEROLS/STEROIDS**	Liebermann-Burchard	++	+++	+++	+++	+++	++	+
	Vanillin-H_3PO_4	+++	++	+++	+++	++	++	++
**IRIDOIDS**	Anisaldehyde- AcAc-H_2SO_4	+	++	++	+	+	-	-
	Vanillin-H_2SO_4	++	+++	+++	+++	++	-	-
**TRITERPENES**	Anisaldehyde- AcAc-H_2SO_4	-	-	-	-	-	-	-
**SAPONINS**		++	+	+++	++	++	++	-
**TRITERPENES AND SAPONINS**	$SbCl_3$/MeOH	++	+++	+++	+++	++	++	-
**SESQUITERPENE LACTONES**	Oleum	-	-	-	-	-	-	-
**ALDEHYDES AND KETONES**	DNPH	++	++	+++	+	++	++	+
**UNSATURATED FATTY ACIDS**	Iodine	+++	+++	+++	+++	+++	+++	++
**ANTIOXIDANT COMPOUNDS**	DPPH	+++	+++	+++	+++	+++	+++	+++

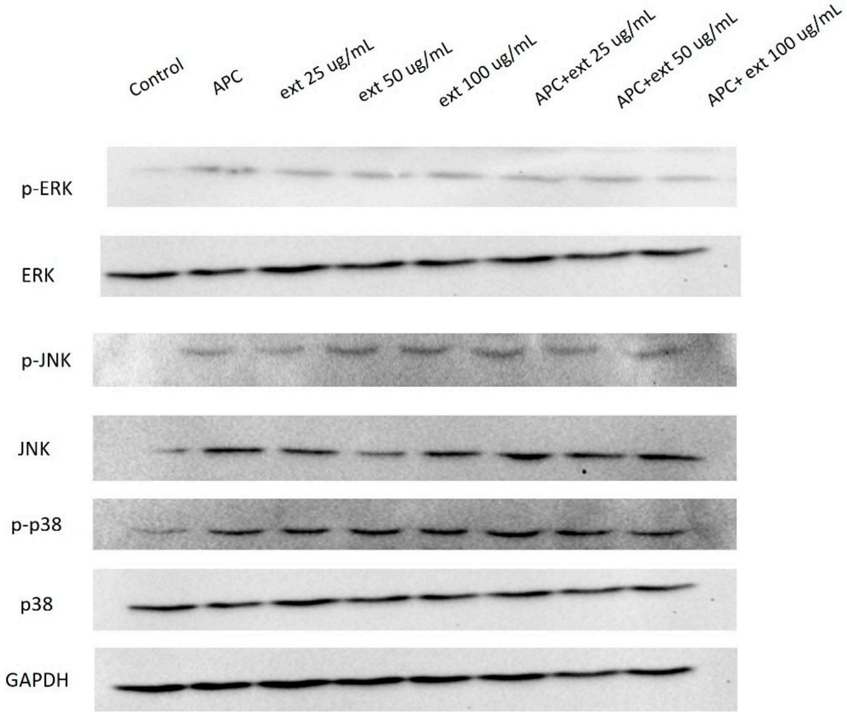

Figure S1. Blotting of MAPK proteins (p38 protein, p.p38, JNK, p-JNK, ERK, p-ERK). in their basal and phosphorylated forms in THP-1 cells treated with the *n*-butanol extract and pretreated with APC for 24 h.

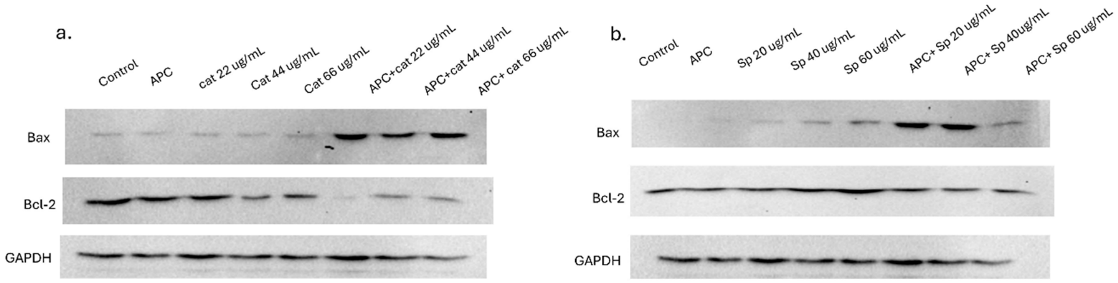

Figure S2. Protein expression of Bcl-2 and Bax in THP-1 cells treated with different concentrations of catalposide (Cat) (a) and specioside (Sp) (b) and pretreated with APC for 24 h, as determined by western blotting.

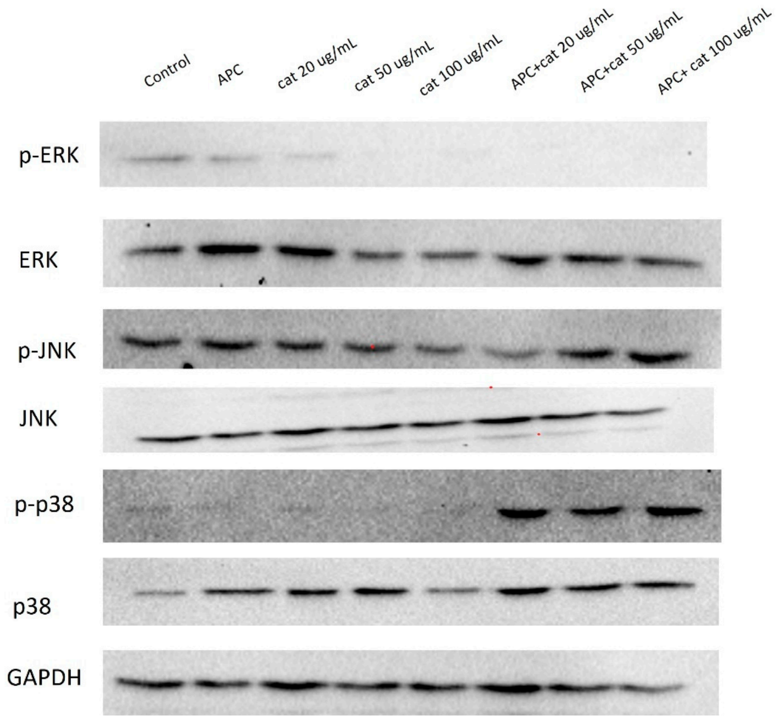

Figure S3. Relative protein expression density (p38 protein, p-p38, JNK, p-JNK, ERK, p-ERK). in their basal and phosphorylated forms in THP-1 cells treated with catalposide (Cat) and pretreated with APC for 24 h.

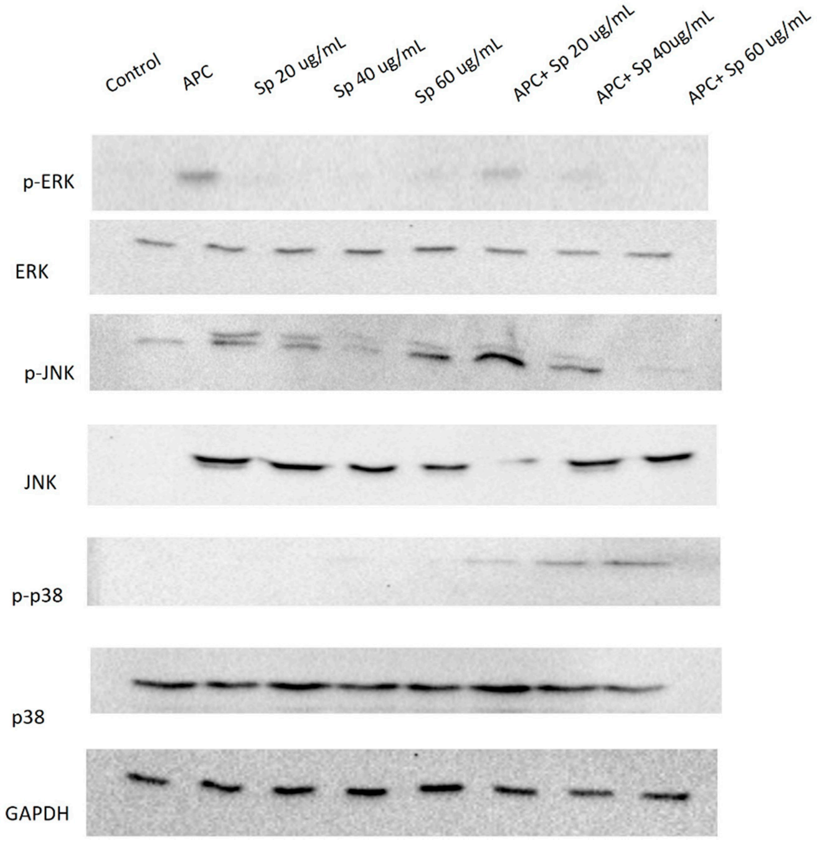

Figure S4. Relative protein expression density (p38 protein, p-p38, JNK, p-JNK, ERK, p-ERK). in their basal and phosphorylated forms in THP-1 cells treated with specioside (Sp) and pretreated with APC for 24 h.

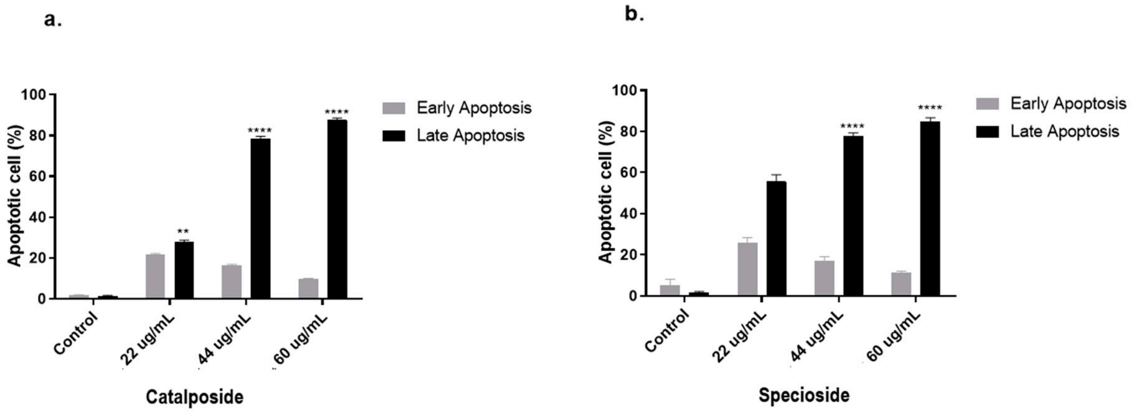

Figure S5. THP-1 cells labeled with Annexin V-CF647 and 7-AAD in early and late apoptosis and treated with different concentrations of Catalposide (a) and Specioside (b) for 24 hours. ** *p≤0.01*,*****p≤0.0001*. ANOVA, Bonferroni post hoc.

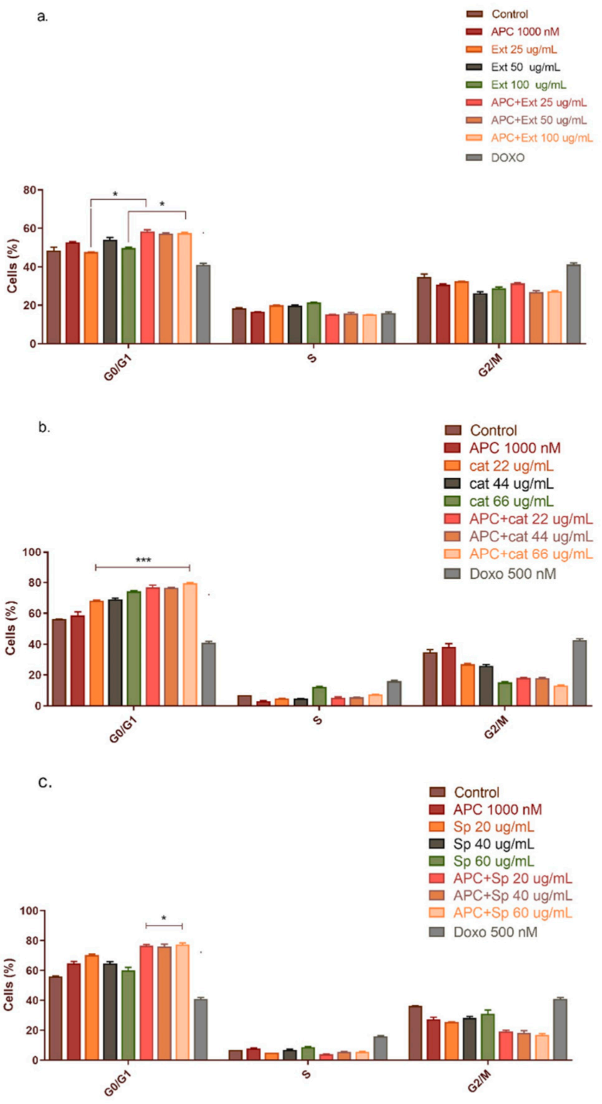

Figure S6. (a) THP-1 cells in each phase of the cell cycle were exposed to the *n*-butanol extract obtained from the inner bark of *T. rosea*. (b) Pretreatment with the catalposide (Cat) and (c) specioside (Sp) and pretreatment with APC for 24 h. *p≤0.05, ***p≤0.001. Kruskal–Wallis, Dunn's post hoc test.

Specioside spectra

The ^{1}H NMR spectrum showed two olefinic protons at δ_H 6.37 (H-3, dd) and δ_H 4.98 (H-4, dd), characteristic of the iridoid nucleus. This structure was confirmed by correlations shown in the HMBC spectrum with carbons at δ_C 140.95 (C-3) and δ_C 101.50 (C-4). In addition, two olefinic protons at δ_H 7.67 (1H, d, *J* = 16.0 Hz, H-7”) and δ_H 6.38 (1H, d, *J* = 15.9 Hz, H-8”) suggested the presence of a *trans* conformation, which is characteristic of a *p*-coumaroyl skeleton. The *p*-coumaroyl structure was confirmed by the observation of two signals at δ_H 7.48 (2H, d, *J* = 8.7 Hz, H-2”, H-6”) and δ_H 6.81 (2H, d, *J* = 8.7 Hz, H-3”, H-5”), characteristic of an AA’XX’ system; these data were confirmed by the ^{13}C NMR spectrum, which exhibited eight carbon signals, including carbonyl carbon δ_C 164.49 (C-9’’), which was attributed to the *p*-coumaroyl ester. The presence of anomeric protons at δ_H 4.79 (1H, d, *J* = 7.9 Hz, H-1’), and methine signals at δ_H 3.42-3.23 (4H, m) are characteristic of a sugar moiety. Analysis of the 1D and 2D NMR spectra in addition to comparisons with literature data for glucoside analogs suggested that the saccharide portion was a glucose moiety. Characteristic ^{1}H NMR, ^{13}C NMR, COSY, HSQC and HMBC spectra are supplied-

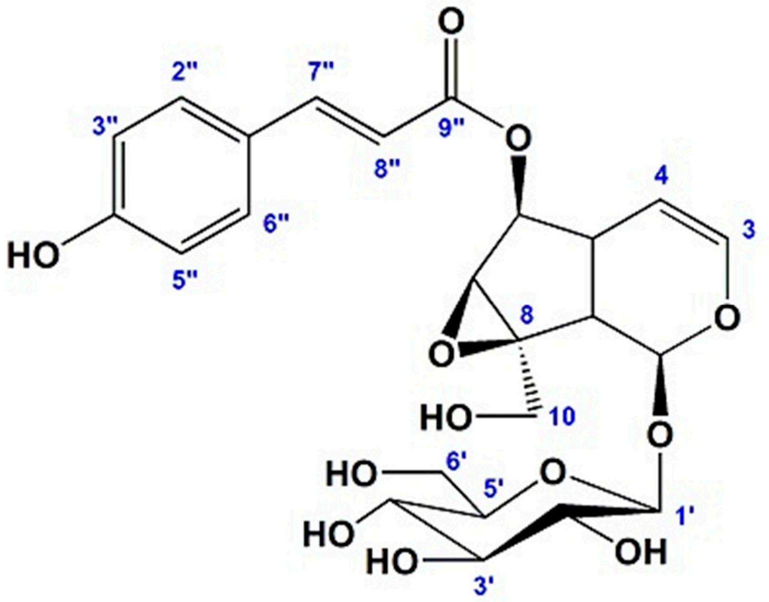

Figure S7. Specioside structure

Figure S8. The ^{1}H NMR spectrum of Specioside in CD3OD, 400 MHz.

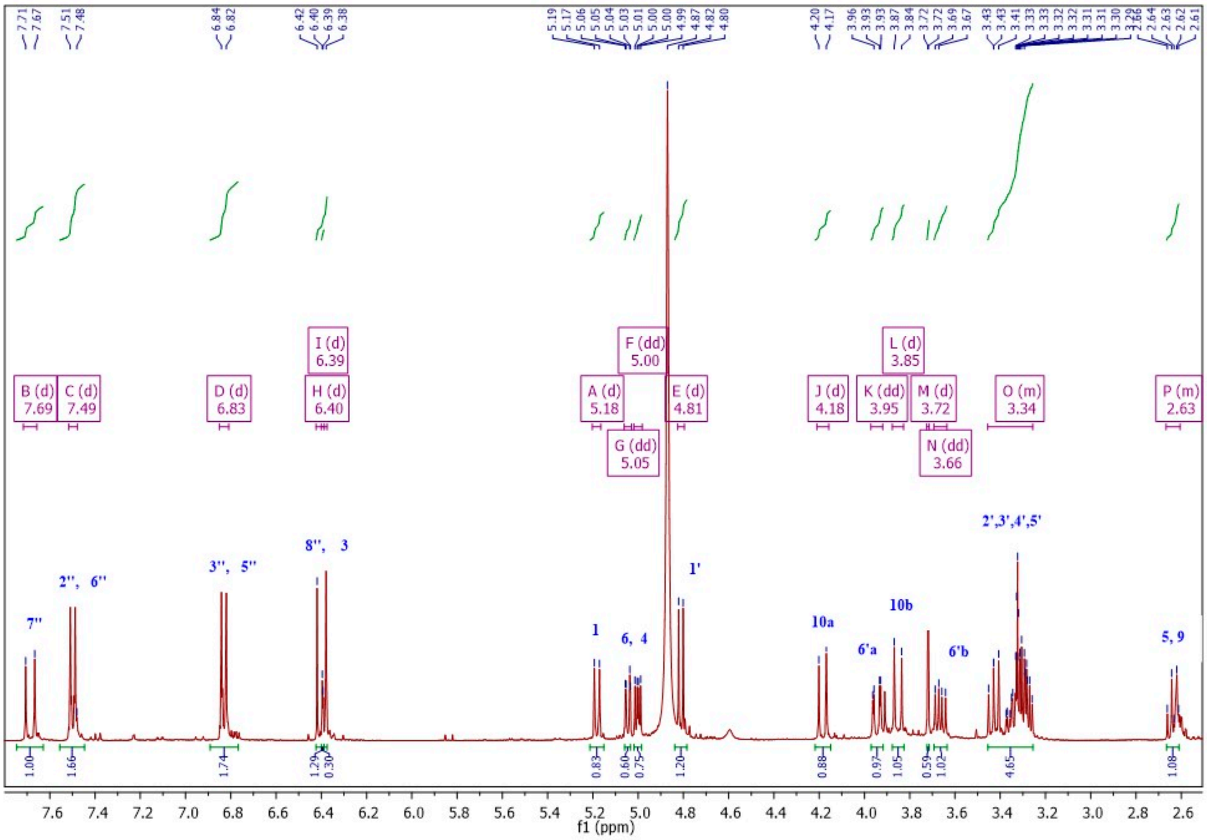

Figure S9. The ^{13}C NMR spectrum of Specioside in CD3OD, 125.6 MHz.

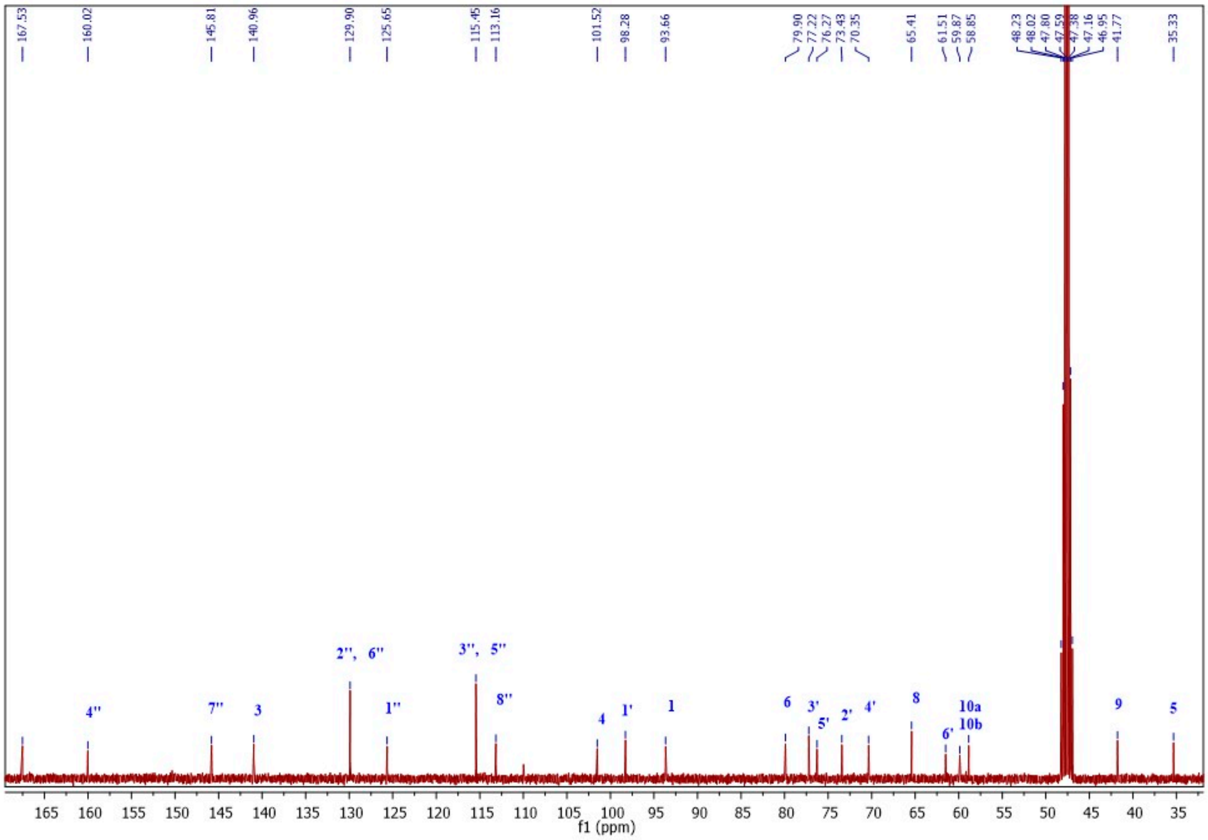

Figure S10. The COSY spectrum of Specioside in CD3OD, 400 MHz.

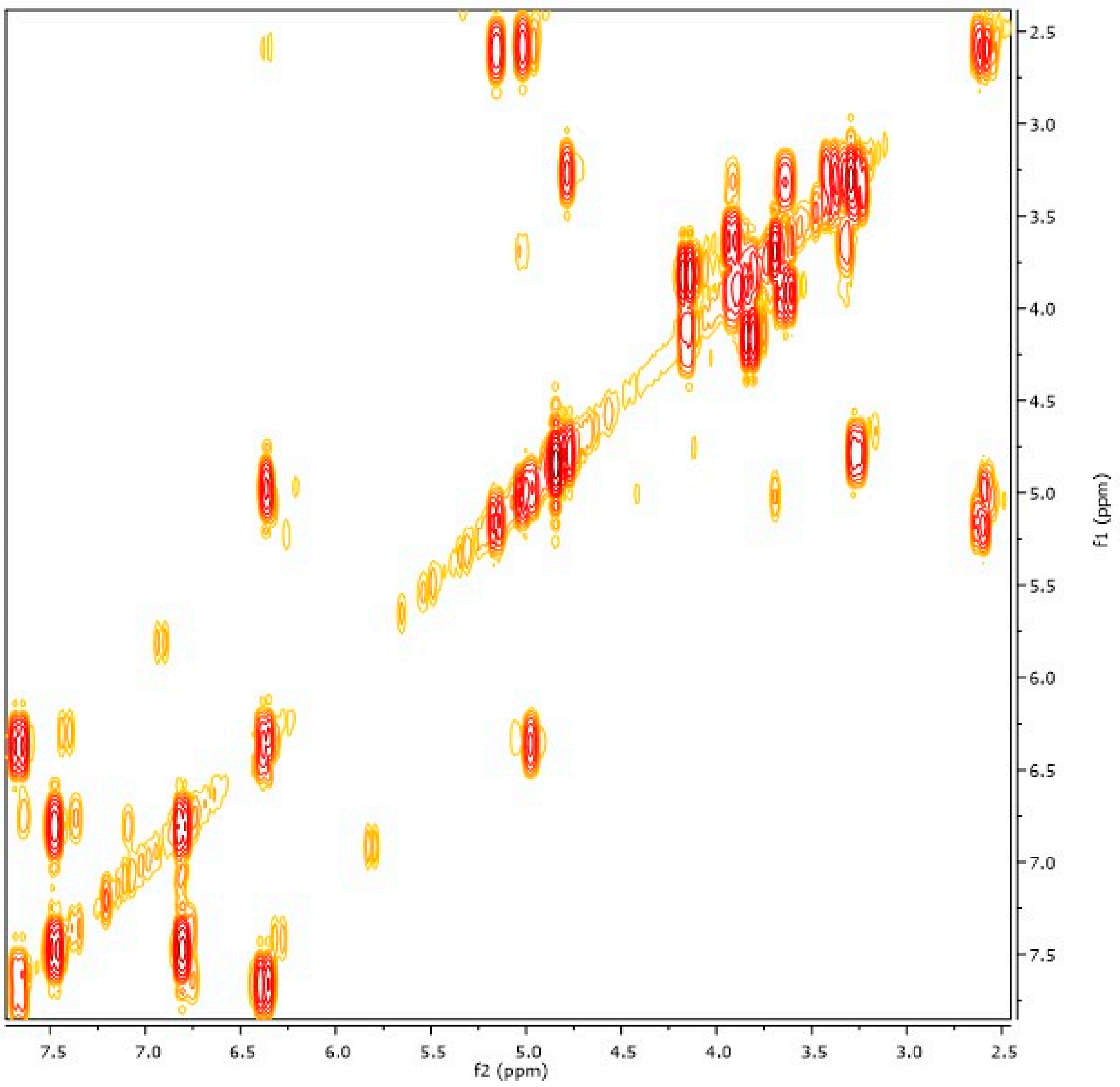

Figure S11. The HSQC spectrum of Specioside in CD3OD, 400 MHz.

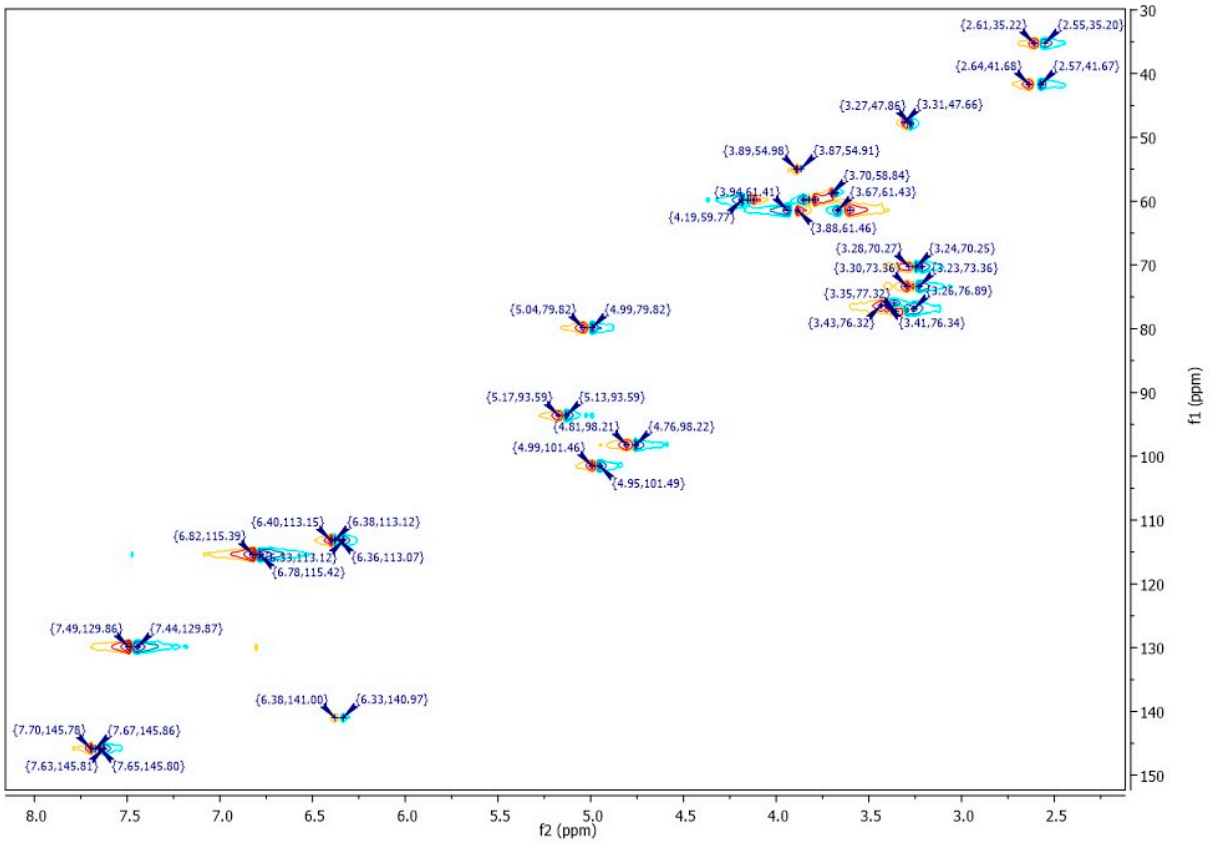

Figure S12. The HMBC spectrum of Specioside in CD3OD, 400 MHz.

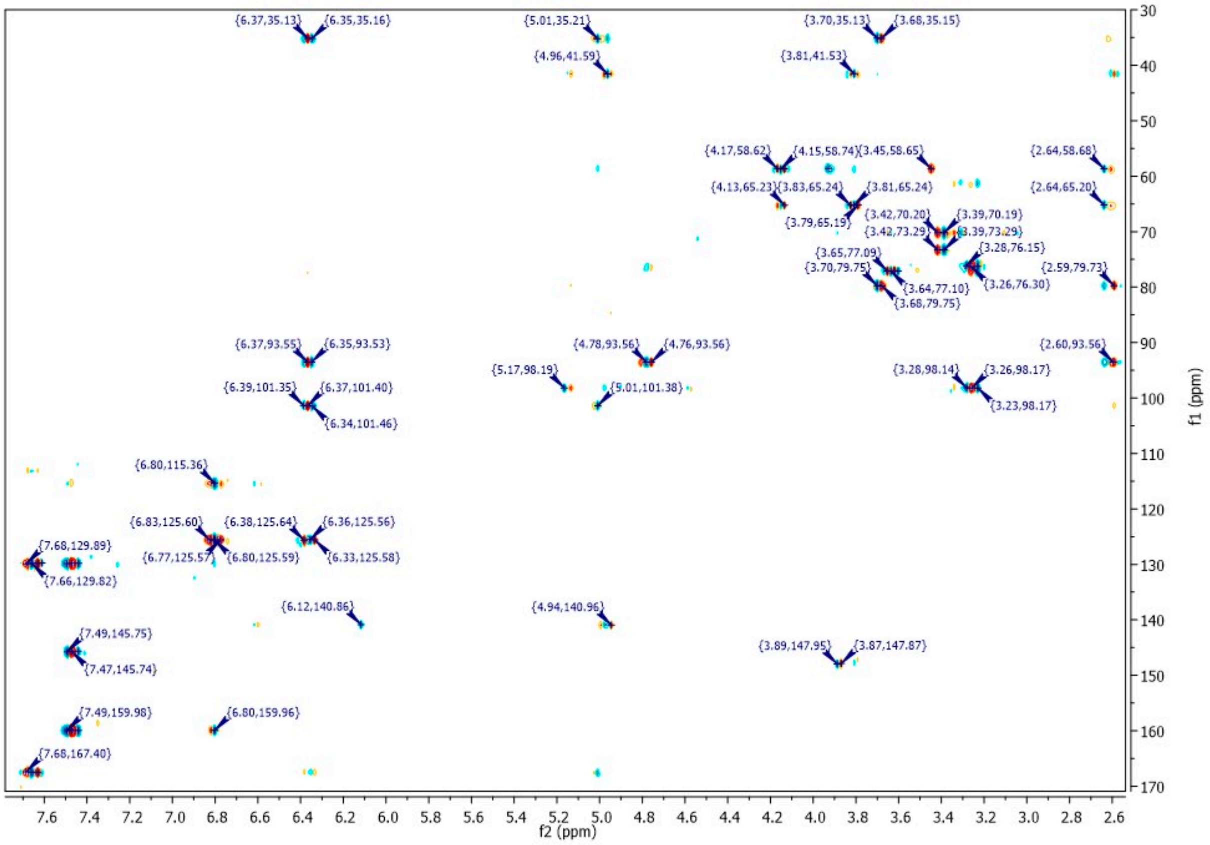

Figure S13. The HMBC expanded spectrum of Specioside (δ 6.4-3.2).

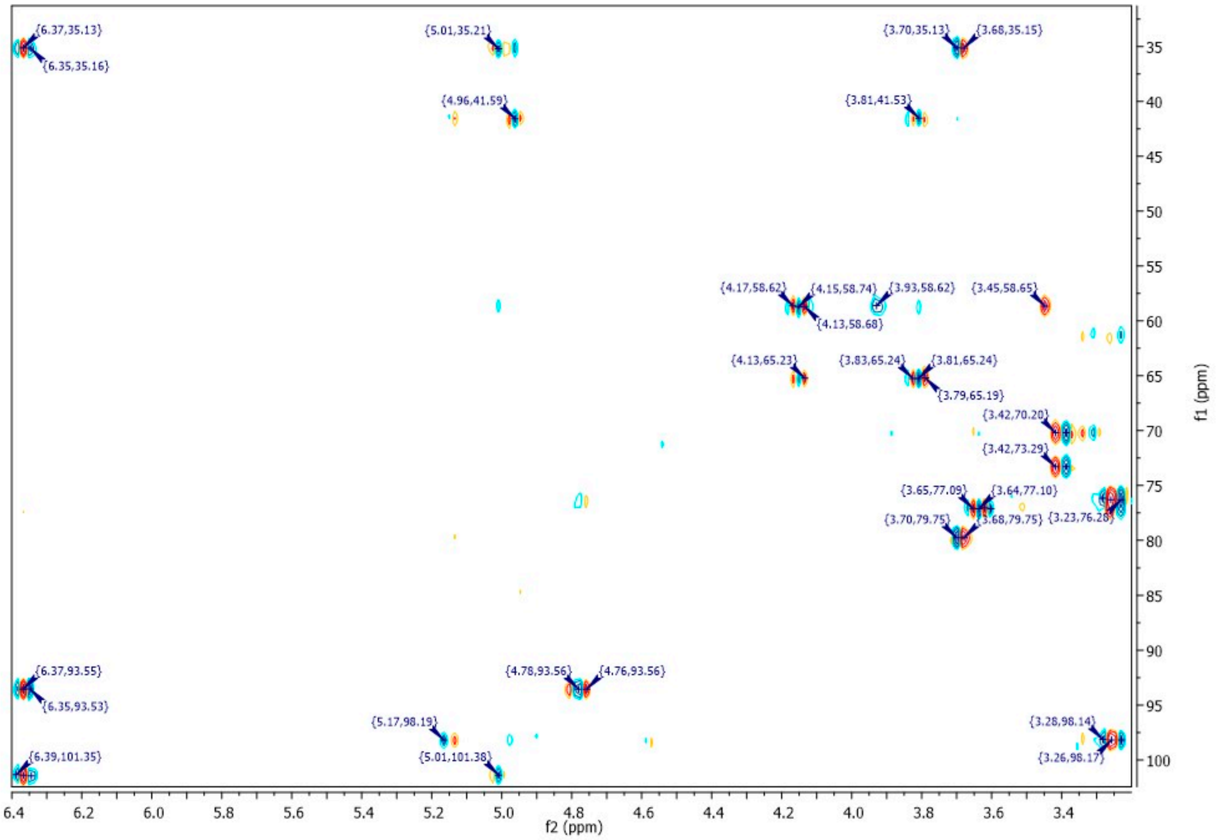

Figure S14. The HMBC expanded spectrum of Specioside (δ 7.8-6.2).

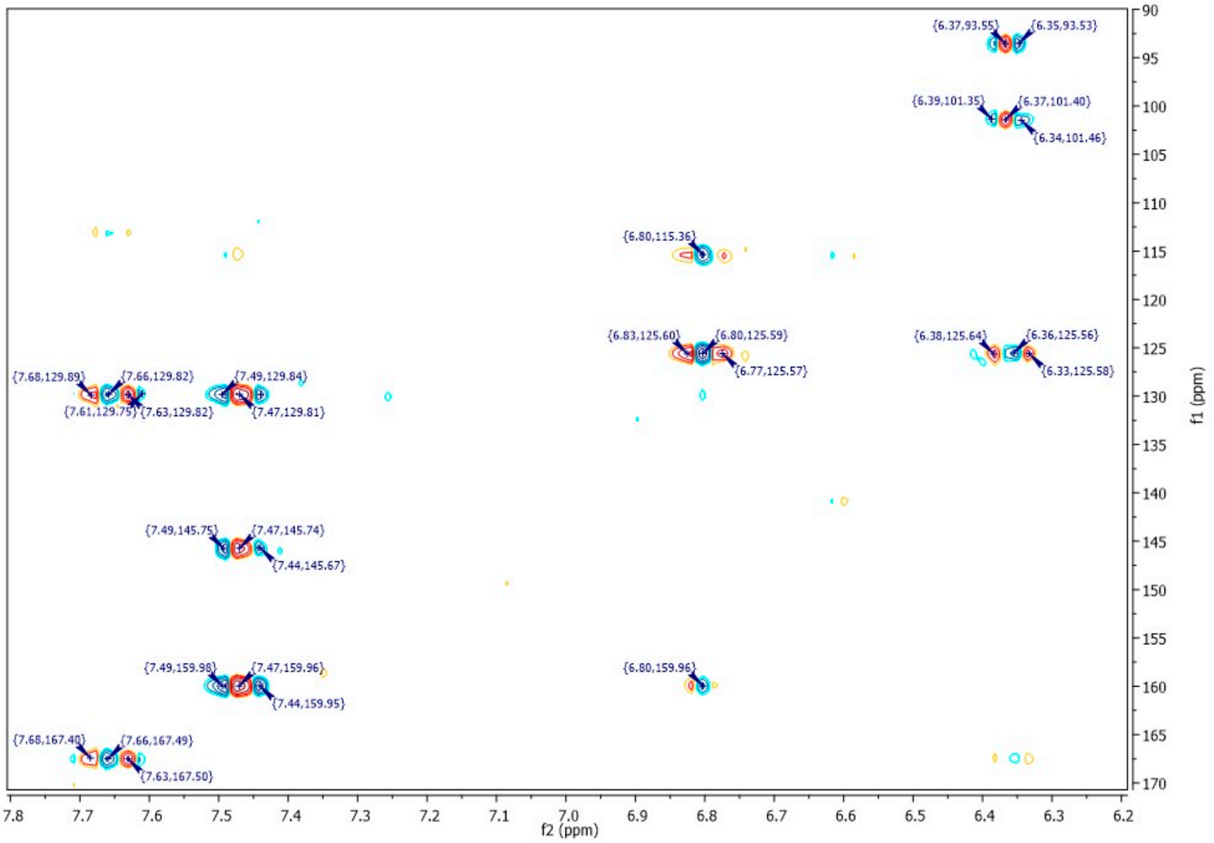

Supplement: Supplementary file 1 [file molecules-29-03986-s001.zip › molecules-3129959-supplementary.pdf]
